# The case for social evaluation in preverbal infants: gazing toward one’s goal drives infants’ preferences for Helpers over Hinderers in the hill paradigm

**DOI:** 10.3389/fpsyg.2014.01563

**Published:** 2015-01-29

**Authors:** J. K. Hamlin

**Affiliations:** Department of Psychology, The University of British ColumbiaVancouver, BC, Canada

**Keywords:** social evaluation, social cognition, helping, hindering, infancy

## Abstract

In a 2007 empirical report, Hamlin, Wynn, and Bloom provided the first evidence that preverbal infants at 6 and at 10 months of age evaluate others on the basis of their helpful and unhelpful actions toward unknown third parties. In their “hill paradigm,” a Climber puppet tried but failed to climb a steep hill, and was alternately bumped up the hill by the Helper and bumped down the hill by the Hinderer. After being habituated to these events, both 10- and 6-month-olds selectively reached for the Helper over the Hinderer. In response, Scarf et al. (2012b) provided evidence that rather than reflecting an early developing capacity for social evaluation, infants’ choices in Hamlin et al. (2007) reflected low-level perceptual preferences whereby infants are drawn to any character who is associated with the Climber bouncing. The current studies represent an attempt to adjudicate between the social and perceptual accounts of infants’ preferences for Helpers over Hinderers in the hill paradigm, by pitting a perceptual cue (e.g., bouncing) against a social cue (e.g., whether or not the Climber gazes toward his goal). Infants’ patterns of preference across two experiments support the social account.

## INTRODUCTION

The ability to distinguish “friend” from “foe” is one of the most important skills for survival in the complex social world. In recent years, a small but growing body of research has reported that the roots of humans’ capacity for social evaluation may be present just a few months after birth. In this research, infants as young as 3 months selectively attend to and reach for prosocial versus antisocial others, preferring individuals who have helped versus prevented others in achieving their unfulfilled goals (see review in Hamlin, 2013a). Evidence to date suggests that within the first year of life, infants socially evaluate characters who help and hinder others in four distinct unfulfilled goal scenarios (Hamlin et al., 2007, 2013a; Hamlin and Wynn, 2011), evaluate helping and hindering flexibly based on the context in which they occur [Hamlin et al., 2011, 2013b; Hamlin, 2014 (but see Scarf et al., 2012a and response by Hamlin et al., 2012)], and evaluate others based on their prosocial and antisocial mental states rather than the positive and negative outcomes they cause (Hamlin et al., 2013a; Hamlin, 2013b). In the second year, infants prefer characters who distribute resources fairly versus unfairly (Geraci and Surian, 2011; see also Burns and Sommerville, 2014), and toddlers selectively direct their own prosocial acts toward helpers and their own antisocial acts toward hinderers (Hamlin et al., 2011). Together, these results suggest that the capacity to distinguish friends from foes is a foundational aspect of humans’ earliest developing social cognitive systems, feeding into emerging systems of sociomoral action.

Despite this growing body of evidence suggestive that young infants prefer prosocial to antisocial others, a recent paper by Scarf et al. (2012b) suggests that early demonstrations of infants’ social evaluations may have been based on physical, rather than social, aspects of helping and hindering events (Scarf et al., 2012b). Specifically, Scarf et al. (2012b) suggest that Hamlin et al.’s (2007; 2010) “hill paradigm” (adapted from original stimuli by Kuhlmeier et al., 2003; see also Premack and Premack, 1997) contained low-level perceptual events that were themselves sufficient to inspire preferences for helpers and against hinderers, without necessitating any unique consideration of the social value of helping and hindering. The current studies represent an attempt to adjudicate between the social and perceptual accounts of infants’ preference for helpers over hinderers in the hill paradigm.

### THE HILL PARADIGM BY Hamlin et al. (2007)

All events began with a “Climber” (most commonly a red, circular wooden character with large plastic ‘googly’ eyes) resting at the bottom of a hill containing two inclines, one shallow and one steep. To imply that the Climber’s goal was to reach the top of the hill, the googly portions of the Climber’s eyes were fixed diagonally upward such that he “gazed” uphill during the entirety of each event^1^. “Helpers” and “Hinderers” were most commonly a blue square and a yellow triangle (whose googly eyes remained moveable); whether the square or the triangle was the Helper was counterbalanced across infants.

At the start of both Helper and Hinderer events, the Climber first moved easily up the shallow incline to a landing where he wiggled back and forth. He then made two unsuccessful attempts to climb the steeper incline; on his first attempt he made it 1/3 of the way up before coming back down and on his second attempt he made it 2/3 of the way. To imply the Climber’s movements up and down the hill reflected a failed intention to climb to the top, during each ascent the Climber decelerated as though fighting upward against gravity, while during each descent he accelerated as though falling down with gravity. On the Climber’s third attempt, Helper and Hinderer events diverged. During Helper events, the Helper entered the scene from the bottom of the hill and bumped the Climber up from below twice, pushing him to the top of the steep incline. The Climber then bounced up and down for several seconds (as though happy to have achieved his goal) and the Helper moved back down the hill and offstage. During Hinderer events, the Hinderer entered the scene from the top of the hill and bumped the Climber down from above twice, forcing him to the bottom of the steep incline. The Climber then rolled end-over-end to the very bottom of the hill and the Hinderer moved back up the hill and offstage. Infants viewed alternating Helper and Hinderer events until a pre-set habituation criterion was reached. Following habituation, infants were presented with the Helper and Hinderer so that they might choose between them. Both 6- and 10-month-olds selectively reached for the Helper, suggesting they had either positively evaluated the Helper, negatively evaluated the Hinderer, or both.

Subsequently, Hamlin et al. (2007) explored the possibility that infants’ preference for Helpers reflected only a perceptually based preference for upward over downward movement, rather than a socially based preference for those who help third parties achieve their goals. New groups of 6- and 10-month-olds viewed stimuli similar to the Helper/Hinderer condition, except that an inanimate, circular red block with no eyes replaced the formerly animate Climber. As self-propelled, non-inertial motion reliably signals agency to infants in this age range (e.g., Premack, 1990; Leslie, 1994, 1995; Rakison and Poulin-Dubois, 2001; Luo and Baillargeon, 2005), the inanimate circle never moved on its own; instead, it was smoothly pushed up the hill by one character and smoothly pushed down the hill by the other. If infants recognize that inanimate blocks cannot possess unfulfilled goals, they should not view these scenes as instances of helping and hindering and so should not prefer a “Pusher-Upper” to a “Pusher-Downer.” Indeed, 6- and 10-month-olds chose Pusher-Uppers and Pusher-Downers at equal rates, suggesting that preferences for Helpers over Hinderers in the animate conditions reflected more than a perceptually based preference for upward over downward movement.

### Scarf et al.’s (2012a) PERCEPTUAL CRITICISM OF THE HILL PARADIGM: IT’S BOUNCING

However, as recently pointed out by Scarf et al. (2012b), upward versus downward movement was not the only perceptual difference between helping and hindering events in Hamlin et al. (2007) stimuli. Specifically, Scarf et al. (2012b) contend that infants approached the Helper because it was associated with the Climber *bouncing* at the top of the hill, and infants perceive bouncing as a positive perceptual event. To test this hypothesis, Scarf et al. (2012b) created their own stimuli and ran a series of studies varying (1) whether the Climber bounced at the end of an event or not, and (2) if there was bouncing, where and when it occurred (at the top or the bottom of the hill, following the intervention of the Helper or the Hinderer). Confirming their hypothesis that bouncing played a role in driving infants’ puppet choices, 10-month-olds consistently reached for *any* character associated with the Climber bouncing, whether it had previously helped or hindered the Climber’s goal. Specifically, when the Climber bounced at the top of the hill after being helped, infants preferred the Helper (replicating Hamlin et al., 2007); but when the Climber bounced at the bottom of the hill after being hindered, infants preferred the Hinderer. When the Climber bounced *both* after being helped and after being hindered, infants chose randomly between the Helper and Hinderer, suggesting that being associated with the act of bouncing is sufficient to make Helpers and Hinderers equally attractive. These results suggest that infants’ preference for Helpers in Hamlin et al.’s (2007) hill paradigm may have been due to low-level perceptual aspects of the hill stimuli, rather than higher-level capacities for social evaluation.

### Hamlin et al.’s (2012) RESPONSE

Hamlin, Wynn, and Bloom were invited to respond to Scarf et al.’s (2012b) perceptual criticism of their work (Hamlin et al., 2012). In their response, Hamlin et al. (2012) noted that there are several *additional* differences between the stimuli created by each group besides bouncing, and that therefore the body of evidence presented to date is insufficient to adjudicate between the social and the perceptual accounts of infants’ preferences in the hill paradigm. First, unlike Hamlin et al.’s (2012) Climber whose gaze was fixed toward the top of the hill, Scarf et al.’s (2012b) Climber had unfixed pupils, which due to gravity and the slope of the hill meant the Climber looked down the hill during much of the procedure. Because gaze direction is a fundamental cue signaling the object of one’s desire in both adults and infants (e.g., Baron-Cohen, 1995; Hood et al., 1998; Pelphrey et al., 2005), this may have prevented infants from understanding that the Climber wished to reach the top of the hill, or even implied he was oriented toward the bottom. Second, while Hamlin et al.’s (2007) Climber varied his speed as he ascended and descended the steep section of the hill, decelerating when moving up and accelerating when moving down to imply struggling and falling, Scarf et al.’s (2012b) Climber moved up and down the hill at uniformly fast rates. This lack of speed variation may have implied that each direction of the Climber’s movement was equally intentional, once again obscuring his goal. Finally, whereas at the end of his final attempt Hamlin et al.’s (2007) Climber moved upward *only* when pushed by the Helper and downward *only* when pushed by the Hinderer, Scarf et al.’s (2012b) Climber continued to move upward between bumps from the Helper (as though he were able to climb the steepest part of the hill on his own) and started moving down the hill before being bumped by the Hinderer (as though he decided to descend, perhaps because he noted the Hinderer was coming down). Hamlin et al. (2012) argued that these three issues might have prevented infants from recognizing the Climber’s goal. If so, Scarf et al.’s (2012b) infants might have chosen puppets based on bouncing simply because they had no reason to interpret the Helper’s and Hinderer’s actions as socially positive or negative.

## THE CURRENT STUDIES

Based on the evidence provided to date, infants’ preference for Helpers in the hill paradigm may reflect considerations of goals, of failed attempts to satisfy those goals, and of actions that facilitate and block such attempts; that is, infants’ choices may reflect social evaluation. On the other hand, infants’ choices may reflect brute perceptual preferences that lead infants to approach anyone associated with an enjoyable physical act such as bouncing. Importantly, stimuli utilized in subsequent studies has not contained such perceptual confounds and infants nevertheless appeared to engage in social evaluation (Hamlin and Wynn, 2011); however, it remains a possibility that infants observing the hill scenario subsequently choose puppets based on low-level perceptual variables rather than higher-level social ones. If infants in the hill scenario do in fact choose puppets based on low-level perceptual variables, it would be important to reconsider the case for social evaluation in infancy.

The current two experiments were created to distinguish between the social and perceptual accounts of infants’ Helper preferences in the hill paradigm. Experiment 1 attempted to replicate both Hamlin et al.’s (2007) and Scarf et al.’s (2012a) findings that infants prefer Helpers over Hinderers, by habituating infants to stimuli videos created by each group (hereafter the *Hamlin condition* and the *Scarf condition*). In Experiment 2, new stimuli were created to directly examine the role of a candidate social cue, whether the Climber gazed uphill, versus a candidate perceptual cue, bouncing, in driving infants’ preference for Helpers. Specifically, in the *No Bounce condition* the Climber’s gaze was fixed uphill, but he did not bounce at the top of the hill after being helped. In contrast, in the *Undirected Gaze condition* the Climber did bounce at the top of the hill after being helped, but his gaze were not fixed uphill.

Because previous research using the hill paradigm has demonstrated the same effect sizes of preference for Helpers over Hinderers throughout the first year (Hamlin et al., 2007, 2010), and to speed data collection, infants between 6- and 11 months of age were tested in all conditions. Analyses of age are reported below; no significant age effects emerged. Verbal and written consent was obtained from infants’ guardians prior to participation. All informed consent and data collection procedures were approved by the University of British Columbia’s behavioral research ethics board (H10-01808) and informed consent was given by the caregivers of all participants.

### EXPERIMENT 1: HAMLIN AND SCARF CONDITIONS

#### Methods

***Participants.*** Forty-eight full-term and typically developing infants between 6- and 11 months of age participated. Twenty-four infants were randomly assigned to the Hamlin condition (12 females; average age = 9 months, 8 days; range = 6;12–11;8), and 24 to the Scarf condition (12 females; average age = 9 months, 1 day; range = 6;16–11;15). An additional 30 infants (11 in Hamlin/19 in Scarf) began or completed the procedure but were not included in the final sample due to fussiness (5/10 infants), procedural error (2/1), technical failure (0/2), failure to choose either puppet (1/4), and parental interference (3/2).

**FIGURE 1:**
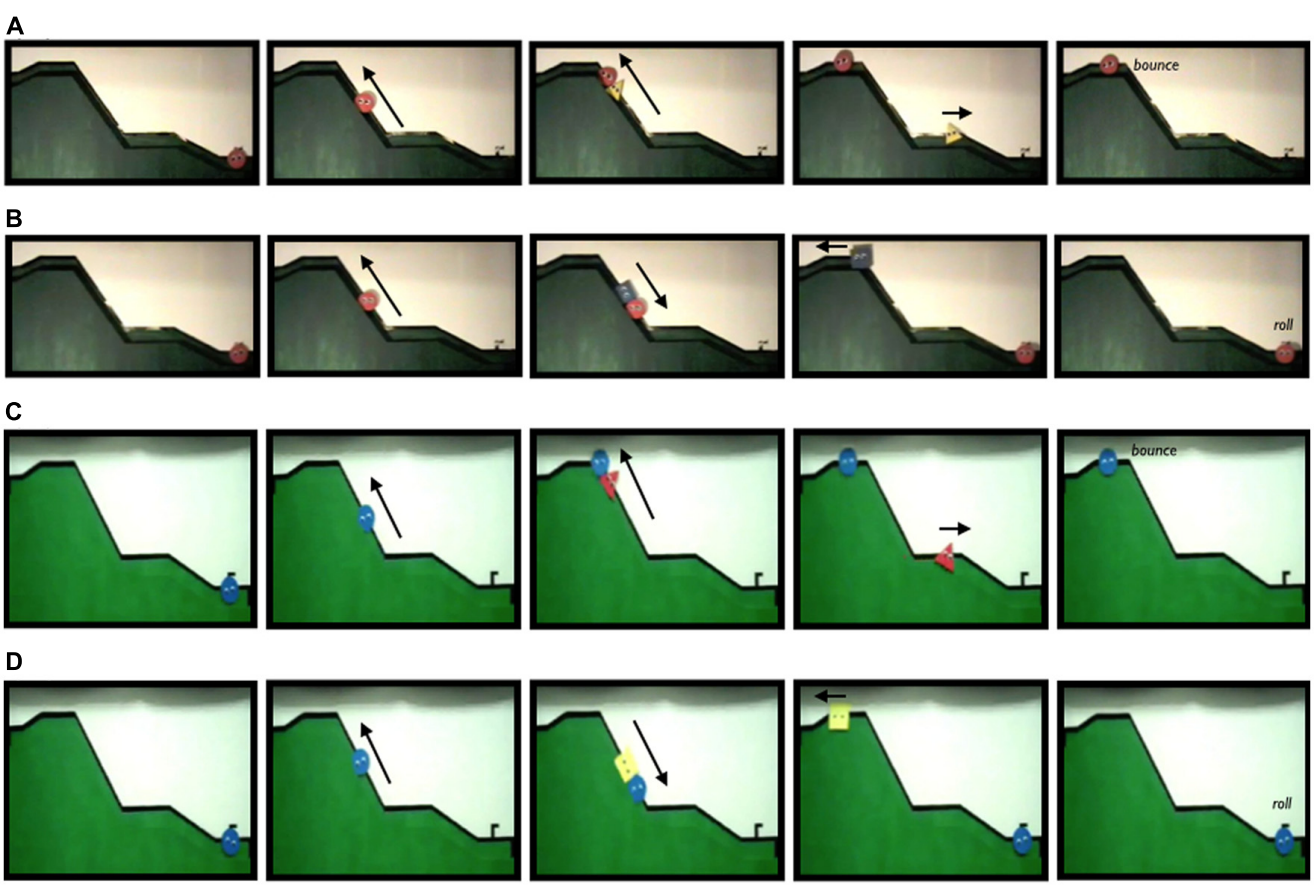
**Screen shots taken from video stimuli presented to infants in Experiment 1’s Hamlin condition (line **A** depicts Helper events, line **B** depicts Hinderer events) and Scarf condition (line **C** depicts Helper events, line **D** depicts Hinderer events)**.

***Procedures.*** Stimuli utilized in the current studies are available at http://cic.psych.ubc.ca/Example_Stimuli.html and are depicted in **Figure 1**. Infants in the Hamlin condition were habituated to the helping and hindering videos that Hamlin et al. (2007) provided as supplementary materials for their 2007 publication^2^, as well as a set of videos in which the color/shape of the Helper and Hinderer were switched (recorded at the same time as the supplementary videos). Thus, in the Hamlin condition the Helper and Hinderer were (counterbalanced across infants) a blue square and a yellow triangle. Infants in the Scarf condition were habituated to the videos that Scarf et al. (2012b) provided as supplementary materials^3^. As only one set of Scarf stimulus videos was available for download, the Helper was always a red triangle and the Hinderer was always a yellow square (experimenters presenting infants with the choice between the Helper and Hinderer were blind to experiment, condition, and puppet identity^4^). Consistent with the differences between the Hamlin et al. (2007) and Scarf et al. (2012a) stimuli detailed above, in both the Hamlin and the Scarf conditions the Climber bounced at the top of the hill after helping, and rolled end-over-end to the bottom of the hill after hindering. In the Hamlin condition the Climber’s gaze was fixed uphill; whereas in the Scarf condition the Climber’s gaze was unfixed. The order of helping and hindering events was counterbalanced across participants in each condition.

Video recordings of hill events were displayed on a large LCD television screen (127 cm diagonal). Infants viewed the videos from their parents’ laps, who were seated in a chair approximately 2 m from the screen. Helper and Hinderer events were presented in alternation until an infant-controlled pre-set habituation criterion was reached, to a maximum of 14 events^5^. A coder viewed infants’ faces on a computer monitor in a room down the hall from the testing room, and recorded infants’ attention toward and away from the display following each event using the program *jHab* (Casstevens, 2007). Coding began from the point the Helper or Hinderer left the stage and the Climber was motionless at the top or bottom of the hill, and continued until infants looked away from the display for two consecutive seconds or until 30 s elapsed. The habituation criterion was met when infants’ summed attention to any three consecutive events starting with the fourth event was less than half their summed attention to the first three events. In subsequent oﬄine attentional coding, attention toward and away from the display during the helping and hindering acts was coded from video recordings acquired during each testing session using *Observer* (Noldus Information Technologies; 3 of 24 videos were unavailable for oﬄine coding in the Scarf condition due to equipment failure).

After habituation, parents were asked to turn their chairs 90° to the left and to situate their infants at the front edge of their laps while grasping them firmly around the lower abdomen, as abdominal support best facilitates reaching behaviors in infancy (e.g., Bertenthal and Von Hofsten, 1998). Parents were instructed to close their eyes, and the experimenter who had coded infants’ attention during habituation (who remained blind to condition/puppet’s identity) entered the testing room and presented infants with a white board on which foam versions of the Helper and Hinderer had been affixed approximately 30 cm apart (side counterbalanced). To present the choice, the experimenter first held the board above the infant’s line of sight and greeted the infant by saying “Hi [baby’s name]!,” ensuring the infant made eye contact. The experimenter then lowered the board down into the infant’s lap so that the characters were in reach, saying “Who do you like?” The infant’s choice was coded online by this experimenter as the first puppet contacted with a visually guided reach (i.e., looking must immediately precede touching). Subsequent reliability coders re-coded 25% of infants’ choices in each Condition from video; there was 100% agreement on puppet choice.

Infants’ choices were compared using non-parametric binomial tests (for within-condition analyses) and Pearson’s chi-square tests (for across-condition analyses). Attention variables, such as rate of habituation and looking time both during and following Helper and Hinderer events in habituation were compared using independent samples *t*-tests and univariate and repeated-measures ANOVAs. Finally, the influence of age and various attention measures on choice were tested using binary linear regressions.

#### Results

***Attention to puppet events.*** Means and standard errors are detailed in **Table 1**. One infant in the Hamlin condition and zero infants in the Scarf condition failed to reach the habituation criterion within 14 events. Infants who did reach habituation in each condition habituated at equal rates [*mean*_Hamlin_ (SEM) = 8.78 (0.66), *mean*_Scarf_ (SEM) = 8.75 (0.51); t_45_ = 0.04, *p* = 0.97; η^2^= 0.00]. Attention to Helper versus Hinderer events during habituation was calculated in two different ways, based on the fact that all infants viewed at least three Helper and three Hinderer events. First, a comparison was made between how long infants attended *after* the first three Helper versus first three Hinderer events, as measured online during the procedure itself. Consistent with our past reports, infants attended equally following Helper and Hinderer events, both within and across each condition (repeated-measures ANOVAs, *F*’s < 1, *p*’s > 0.69), suggesting that 6–11-month-olds have no baseline assumptions about individuals’ relative likelihood to help and to hinder third parties (but see Geraci and Surian, 2011; Schmidt and Sommerville, 2011; Sloane et al., 2012, for evidence with toddlers’ expectations of fair/unfair distributions). Second, a comparison was made between how long infants attended *during* the first three Helper versus the first three Hinderer events, as measured oﬄine from video. Infants attended equally during Helper and Hinderer events, both within and across condition (repeated-measures ANOVAs, *F*’s < 1, *p*’s > 0.48).

**Table 1:** Average attention during and after the first three Helper and Hinderer events in each condition in Experiment 1.

	# Hab trials	Attention during first three helper events	Attention during first three hinderer events	Attention after first three helper events	Attention after first three hinderer events
Hamlin Condition	8.78 (0.66)	10.83 (0.48)	10.70 (0.44)	6.79 (0.86)	6.50 (0.80)
Scarf Condition	8.75 (0.51)	10.39 (0.54)	10.67 (0.75)	6.77 (0.85)	6.87 (1.01)

*Numbers in parentheses are SEs.*

**FIGURE 2:**
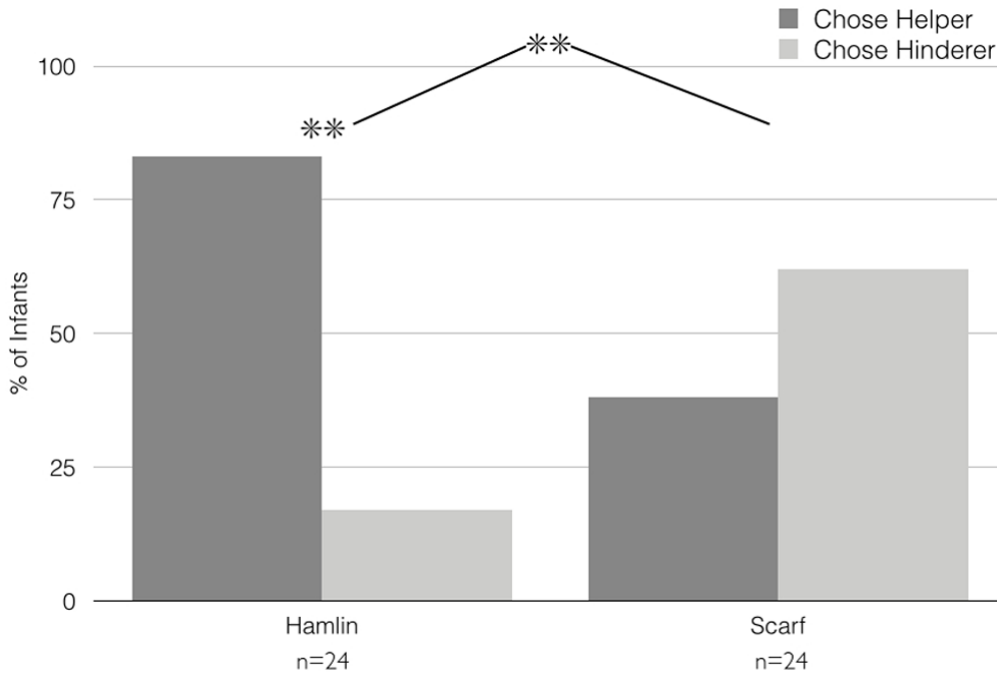
**Results, Experiment 1.** Infants’ choices of the Helper versus the Hinderer in the Hamlin and Scarf conditions of Experiment 1. **p < 0.005.

***Choice.*** Choices are depicted in **Figure 2**. Non-parametric analyses of infants’ preference for Helpers versus Hinderers revealed that infants in the Hamlin and Scarf conditions preferred Helpers at significantly different rates (Pearson’s χ^2^ (*df* = 1) = 10.54, *p* = 0.001). Specifically, infants in the Hamlin condition significantly preferred the Helper to the Hinderer (20 of 24, binomial *p* = 0.002), whereas infants in the Scarf condition were equally likely to prefer Helpers and Hinderers (9 of 24 chose the Helper; binomial *p* = 0.31). Subsequent chi-square tests revealed no effects of infant gender, order of Helper/Hinderer events during habituation, shape/color of Helper puppet, or side of Helper puppet during choice on infants’ choices within or across conditions.

To examine whether attention during or after Helper and Hinderer events in habituation influenced an infant’s tendency to choose the Helper during choice, binary logistic regressions on choice were performed within each condition attention during and after the first three Helper and Hinderer events as covariates. No significant effects emerged (binary logistic regression, -0.55 < coefficients < 0.33, *p*’s > 0.24). To examine whether infants’ age affected their tendency to choose the Helper in either condition, a binary logistic regression on choice was conducted within each condition with age as a covariate; there was no effect of age in the Hamlin condition (binary logistic regression, coefficient = -0.71, *p* = 0.18) and no effect of age in the Scarf condition (coefficient = 0.062, *p* = 0.84).

#### Summary and Discussion

Infants in Experiment 1 were habituated to videos downloaded from the supplementary materials accompanying Hamlin et al. (2007) and Scarf et al. (2012b). Choice results in the Hamlin condition replicated the results of Hamlin et al. (2007), but choices in the Scarf condition failed to replicate the results of Scarf et al. (2012b). Specifically, infants were significantly more likely to reach for the Helper versus the Hinderer in the Hamlin condition, when the Climber’s eyes pointed up the hill toward his goal, but were no more likely to reach for the Helper versus the Hinderer in the Scarf condition, when the Climber’s eyes were unfixed. Because the Climber bounced at the top of the hill following helping acts in both the Hamlin and Scarf conditions, these results call into question Scarf et al.’s (2012b) suggestion that bouncing alone is sufficient to drive infants’ preferences for Helpers over Hinderers in the hill paradigm.

However, as outlined in section 1.3 of the introduction, the original stimuli from Hamlin et al. (2007) and Scarf et al. (2012b) contain several differences in addition to fixed versus unfixed gaze, including variations in the Climber’s speed upon ascending and descending the hill and whether or not the Helper and Hinderer are uniquely responsible for pushing the Climber to the top and bottom. These differences make it difficult to determine exactly why infants preferred Helpers to Hinderers in the Hamlin but not in the Scarf conditions: between-condition differences may have been due to fixed versus unfixed eye gaze, or they may have stemmed from other uncontrolled aspects of the displays. These differences, as well as the difference in Helper and Hinderer shape/color between the Hamlin and Scarf conditions and the inability to counterbalance Helper identity in the Scarf condition, make Experiment 1 an imperfect test of the social versus perceptual accounts of infants’ Helper preferences. Furthermore, results from Experiment 1 do not effectively clarify the role of bouncing in infants’ positive evaluations of Helpers in the hill paradigm: although results suggest that a bouncing event is not *sufficient* to inspire positive evaluation of Helpers (as there was bouncing in the Scarf condition but infants showed no systematic preferences), it may nevertheless be *necessary* for them. For instance, infants in the Hamlin condition may have viewed bouncing as the Climber’s positive reaction upon achieving his goal, further reinforcing that he had intended to climb the hill, and highlighting the Helper’s role in facilitating that intention. Experiment 2 was designed to distinguish between these possibilities.

### EXPERIMENT 2

Experiment 2 was run concurrently with Experiment 1, and was designed to provide a more stringent test of the role of both bouncing and uphill gaze in driving infants’ preference for Helpers in the hill paradigm. New stimuli were created by filming puppet shows performed on a wooden hill apparatus identical to the foam core apparatus utilized by Hamlin et al. (2007) and Scarf et al. (2012b) (but note that the hill itself was somewhat lighter in color). In the No Bounce condition, the Climber’s gaze was fixed uphill, but he did not bounce upon reaching the top of the hill during Helper events, nor did he roll end-over-end to the bottom of the hill during Hinderer events. In the Undirected Gaze condition, the Climber’s gaze was not fixed upward, but he did bounce upon reaching the top of the hill during Helper events and did roll end-over-end to the bottom of the hill during Hinderer events. All other aspects of the stimuli were identical across conditions: the Climber decelerated upon ascending the hill and accelerated upon descending, and during his final attempt moved to the top and bottom of the hill solely through bumps from the Helper and Hinderer. If the social evaluation account of infants’ Helper preferences is correct, in Experiment 2 infants in the No Bounce condition should prefer the Helper but infants in the Undirected Gaze condition should not. On the other hand, if the perceptual evaluation account is correct, infants in the Undirected Gaze condition should prefer the Helper but infants in the No Bounce condition should not.

#### Methods

***Participants.*** Fifty full-term and typically developing infants between 6- and 11 months of age participated. Twenty-five infants were randomly assigned to the *No Bounce condition* (12 females; average age = 9 months, 4 days; range = 6;17–11;13), and 25 to the *Undirected Gaze condition* (13 females; average age = 9 months, 10 days; range = 6;15–11;10). An additional 23 infants (8 in No Bounce/15 in Undirected Gaze) began or completed the procedure but were not included in the final sample due to fussiness (4/7 infants), procedural error (2/4), technical failure (1/1), failure to choose either puppet (1/2), and parental interference (0/1). Conditions in Experiment 2 contained one more baby per condition than conditions in Experiment 1 because more babies were scheduled than were needed; removing the last baby from each condition in Experiment 2 reveals the exact same pattern of results. Due to a period of lab construction, one infant in the Undirected Gaze condition was tested in an alternative space with a smaller screen (81 cm diagonal). This participant was seated closer to the screen (∼1.2 m away). This infant chose the Helper puppet; removing this participant from the sample does not influence the results.

**FIGURE 3:**
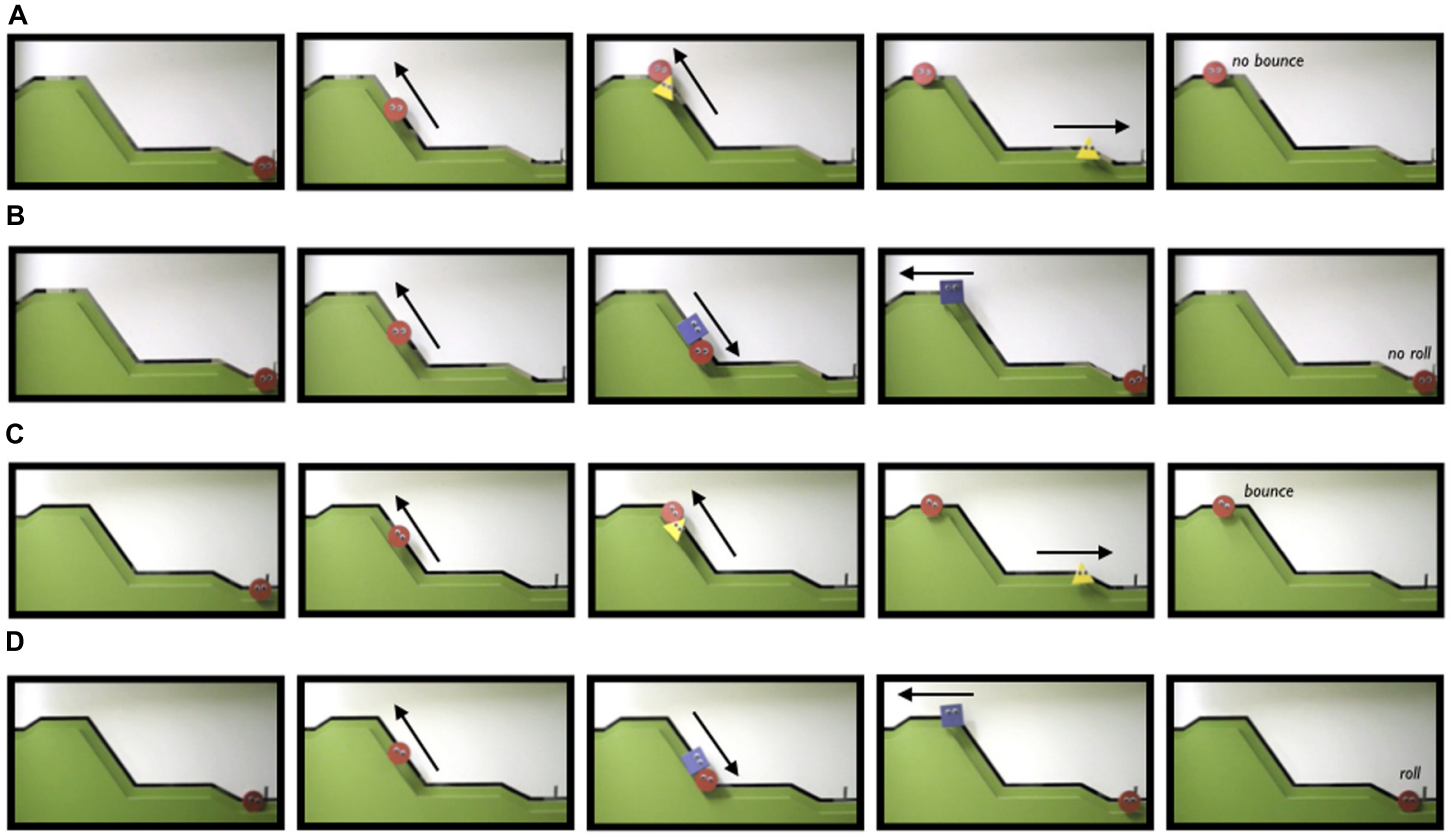
**Screen shots taken from video stimuli presented to infants in Experiment 2’s No Bounce condition (line **A** depicts Helper events, line **B** depicts Hinderer events) and Undirected Gaze condition (line **C** depicts Helper events, line **D** depicts Hinderer events)**.

***Procedures.*** Stimuli are available at http://cic.psych.ubc.ca/Example_Stimuli.html and are depicted in **Figure 3**. In both the No Bounce and Undirected Gaze conditions the Helper and Hinderer puppets were a yellow triangle and a blue square; Helper identity was counterbalanced across infants within each condition. All attentional coding, choice, and data analysis procedures are identical to those of Experiment 1. Reliability coders re-coded 25% of infants’ choices in each condition; there was 100% agreement on puppet choice.

#### Results

***Attention to puppet events.*** Means and SE are detailed in **Table 2**. One infant in the No Bounce condition and two in the Undirected Gaze condition failed to reach the habituation criterion within 14 events. Infants who did reach habituation habituated at equal rates [*mean*_NoBounce_ (SEM) = 8.58 (0.52), *mean*_UndirectedGaze_ (SEM) = 8.09 (0.39); *t*_45_ = 0.76, *p* = 0.45; η^2^= 0.01]. As in Experiment 1, a repeated-measures ANOVA on infants’ attention *following* Helper and Hinderer events with condition as a between-subjects factor revealed no significant effects or interactions (repeated-measures ANOVAs, *F*’s < 1, *p*’s > 0.55). Unlike in Experiment 1, however, there was a significant main effect of condition on infants’ attention *during* Helper and Hinderer events (*F*_1,44_ = 11.13; *p* = 0.002, $\eta_{p}^{2}$ = 0.20), reflecting that infants attended significantly longer during Helper events and marginally longer during Hinderer events in the No Bounce condition than in the Undirected Gaze condition [*mean*_HelperNB_ (SEM) = 12.06 (0.25), *mean*_HelperUG_ (SEM) = 10.49 (0.31), *F*_1,44_ = 15.78, *p* < 0.001, $\eta_{p}^{2}$ = 0.26; *mean*_HindererNB_ (SEM) = 11.60 (0.12), *mean*_HindererUG_ (SEM) = 10.51 (0.56); *F*_1,44_= 3.56, *p* = 0.07, $\eta_{p}^{2}$ = 0.08]. In addition, within the No Bounce condition specifically infants attended marginally longer during Helper events than during Hinderer events (*F*_1,22_ = 3.50, *p* = 0.08, $\eta_{p}^{2}$ = 0.14; note that the average difference is less than half of a second); whereas infants in the Undirected Gaze condition attended equally during Helper and Hinderer events (*F*_1,22_= 0.002, *p* = 0.97, $\eta_{p}^{2}$ = 0.00). These attentional differences will be returned to below.

**Table 2:** Average attention during and after the first three Helper and Hinderer events in each condition in Experiment 2.

	# Hab trials	Attention during first three helper events	Attention during first three hinderer events	Attention after first three helper events	Attention after first three hinderer events
No Bounce condition	8.58 (0.52)	12.06 (0.25)^1,a^	11.60 (0.12)^1,2^	7.38 (0.90)	7.12 (1.05)
Undirected Gaze condition	8.09 (0.39)	10.49 (0.31)^a^	10.51 (0.56)^2^	7.66 (0.77)	7.22 (1.15)

*Numbers in parentheses are SEs. Boxes containing the same letters are significantly different from each other (*p* < 0.001); boxes containing the name numbers are marginally different from each other (*p* < 0.08).*

**FIGURE 4:**
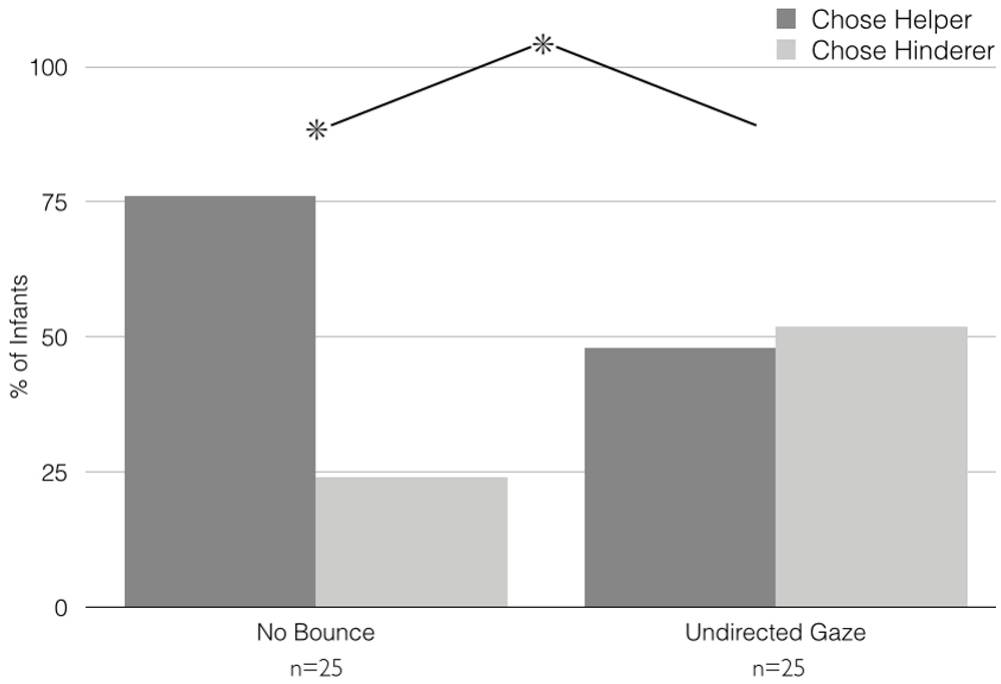
**Results, Experiment 2.** Infants’ choices of the Helper versus the Hinderer in the No Bounce and Undirected Gaze conditions of Experiment 2. *p < 0.05.

***Choice.*** Choices are depicted in **Figure 4**. Non-parametric analyses of infants’ preference for Helpers versus Hinderers revealed that infants in the No Bounce and Undirected Gaze conditions preferred Helpers at significantly different rates [Pearson’s χ^2^ (*df* = 1) = 4.16, *p* = 0.04]. Specifically, infants in the No Bounce condition significantly preferred the Helper to the Hinderer (19 of 25, binomial *p* = 0.01), whereas infants in the Undirected Gaze condition were equally likely to prefer Helpers and Hinderers (12 of 25 chose the Helper; binomial *p* = 1). As in Experiment 1, additional chi-square tests revealed no effects of infant gender, order of Helper/Hinderer events during habituation, shape/color of Helper puppet, or side of Helper puppet during choice on infants’ choices within or across conditions.

As in the Hamlin and Scarf conditions of Experiment 1, there was no effect of age on infants’ tendency to choose the Helper in the No Bounce condition (binary logistic regression, coefficient = 0.14, *p* = 0.63) nor in the Undirected Gaze condition (coefficient = 0.22, *p* = 0.48). Although attentional differences during (but not after) Helper and Hinderer events emerged both across and within conditions in Experiment 2, it is unlikely that these differences are responsible for between-condition differences in infants’ tendency to prefer Helpers to Hinderers. First, as in Experiment 1, binary logistic regressions on choice with attention both during and after Helper events and during and after Hinderer events as covariates revealed no effects of any covariate in either condition (-0.27 < coefficient < 0.63, *p*’s > 0.13), suggesting that attending more during particular events during habituation did not influence individual infants’ tendency to choose the Helper rather than the Hinderer. Furthermore, infants in the Hamlin and Scarf conditions of Experiment 1 and infants in the Undirected Gaze condition of Experiment 2 attended equally during all events, but showed a significant preference for Helpers over Hinderers only in the Hamlin condition. Together, these results suggest that it was whether or not the Climber demonstrated a clear unfulfilled goal, and not differences in attention to particular events or conditions, that drove infants’ patterns of choice across conditions.

## GENERAL DISCUSSION

Across Experiments 1 and 2, 6–11-month-olds’ pattern of preference for Helpers over Hinderers suggests that they evaluate characters based on their relative social value, rather than on whether they happen to be associated with bouncing. Specifically, when the Climber’s gaze was *consistent* with the goal of reaching the top of the hill (in the Hamlin condition in Experiment 1 and in the No Bounce condition in Experiment 2) infants selectively reached for the Helper who pushed the Climber up the hill over the Hinderer who pushed him down. In contrast, when the Climber’s gaze was *inconsistent* with the goal of reaching the top of the hill (in the Scarf condition in Experiment 1 and in the Undirected Gaze condition in Experiment 2) infants chose randomly between the characters. Critically, the Climber bouncing upon reaching the top of the hill was neither necessary, nor sufficient, for infants to engage in social evaluation: 19 of 25 infants (76%) preferred the Helper in the No Bounce condition in which no bouncing occurred, but only 21 of 49 (43%) preferred the Helper in the Scarf and Undirected Gaze conditions, though bouncing occurred in both. It was also not the case that bouncing somehow *prevented* infants from engaging in social evaluation by distracting them from their preference for the Helper: 20 of 24 (83%) infants in the Hamlin condition in which the Climber gazed uphill preferred the Helper, even though the Climber bounced upon reaching the top. Together, this pattern of results contradicts Scarf et al.’s (2012b) lower-level perceptual account of infants’ choices and supports Hamlin et al.’s (2007) higher-level social account: infants’ choices appear sensitive to the basic notion that an action is only helpful or unhelpful to the extent that it facilitates or blocks someone’s goal. These results are consistent with a growing body of work suggesting that infants’ evaluations are selective to the actions of social agents toward social agents, flexible based on the context in which helpful and unhelpful actions occur, and focused specifically on helpers’ and hinderers’ mental states (see Hamlin, 2013a for review).

The critical nature of gaze in the current studies is consistent with a large literature highlighting the role of gaze in understanding others’ goals, both in typically developing and autistic individuals (e.g., Baron-Cohen, 1995; Baron-Cohen et al., 1995; Hood et al., 1998; Johnson et al., 1998; Pelphrey et al., 2005). Although various movement cues (for example, speed acceleration/deceleration) may *also* have clouded infants’ understanding of the Climber’s goals in the Scarf condition, the current studies were specifically designed to address Scarf et al.’s (2012b) contention that infants’ preference for Helpers is solely due to bouncing as well as to highlight the role of uphill gaze. Because isolating whether movement cues play a unique role in infants’ goal perception was outside the scope of this work, the Climber moved as though he intended to climb the hill in both conditions in Experiment 2. That infants did not prefer the Helper in the Undirected Gaze condition despite the presence of goal-directed movements suggests that gaze direction might be a relatively stronger indicator of another’s goal than are their movements. If so, future studies might examine the role of goal-directed movement in infants’ social evaluations by utilizing characters whose movements do or do not appear directed toward the goal of reaching the top of the hill, but who have no eyes at all (see, e.g., Kuhlmeier et al., 2003; Wagner and Carey, 2005; see also Heider and Simmel, 1944).

Beyond distinguishing between the social and perceptual accounts of infants’ puppet choices in the hill paradigm specifically, there are significant theoretical reasons to continue to explore these issues. In particular, Hamlin et al. (2007) raise the possibility that “the capacity to evaluate individuals on the basis of their social interactions is universal and unlearned” (Hamlin et al., 2007; quoted in Scarf et al., 2012b). This is a theoretical claim that, if true, would call for a partial revision of some of our most basic assumptions about the way complex social capacities develop (e.g., Carpendale and Lewis, 2006). Therefore, it is no wonder that developmental researchers like Scarf et al. (2012b) have been motivated to examine the accuracy of data purporting to demonstrate high-level capacities in pre-verbal infants, and wish to “speak more generally to the issue of rich interpretations of infant behavior.” Indeed, there is a long debate between developmental scientists about how best to interpret necessarily simple infant responses such as looking time or preference behaviors. Whereas some are willing to attribute rich cognitive bases to such behaviors as long as there are control conditions to rule out some of the more plausible low-level interpretations, others criticize all rich interpretations on the grounds that lower-level explanations are always possible and always preferable (for examples from both sides see Premack, 1990; Leslie, 1994; Spelke, 1994, 1998; Haith, 1998; Aslin, 2000; Bogartz et al., 2000; Cohen and Marks, 2002; Wynn, 2002; Kagan, 2008; Ruffman et al., 2012; Scarf et al., 2012a,b; Margolis and Laurence, 2013). That said, whatever the current evidence for and against either theoretical stance, to the extent that such debate promotes rigorous empirical efforts to distinguish between alternatives it can only benefit the field as a whole.

## Conflict of Interest Statement

The author declares that the research was conducted in the absence of any commercial or financial relationships that could be construed as a potential conflict of interest.

## Footnotes

1 I regret that this methodological detail was omitted from the methods sections of Hamlin et al. (2007, 2010). It was, however, observable in the stimuli videos available online.

2 http://www.yale.edu/infantlab/socialevaluation

3 http://www.youtube.com/user/DamianScarfPhD/videos

4 Because the Helper/Hinderer were of an atypical color/shape combination in the Scarf condition, some more observant research assistants may have inferred that an infant was in the Scarf condition when they presented the choice. It is unlikely, however, that this could have lead to any systematic biases in the pattern of results. First, experimenters were unaware that identity was not counterbalanced in the Scarf condition, and since identity is counterbalanced in every single other study in the laboratory, it seems unlikely anyone would infer that it was not in this condition alone. Second, research assistants are frequently reminded of the importance of experimenter blinding, and know that they must quickly identify any situation in which they become un-blinded to which puppet is which during a study so that a new experimenter can be switched in; no experimenters reported detecting the lack of identity counterbalancing in the Scarf condition. Finally, multiple different experimenters (10 or more per condition) presented choice for these studies, making it unlikely that any one individual could have come to this knowledge via performing several choices in the Scarf condition.

5 Note: Scarf et al. (2012b) used 10 total events, and Hamlin et al. (2007) used 14.
